# Comprehensive Evaluation of microRNA Expression Profiling Reveals the Neural Signaling Specific Cytotoxicity of Superparamagnetic Iron Oxide Nanoparticles (SPIONs) through N-Methyl-D-Aspartate Receptor

**DOI:** 10.1371/journal.pone.0121671

**Published:** 2015-03-23

**Authors:** Bo Sun, Rui Liu, Nan Ye, Zhong-Dang Xiao

**Affiliations:** 1 State Key Laboratory of Bioelectronics, School of Biological Science and Medical Engineering, Southeast University, Nanjing 210096, P. R. China; 2 Institute of Microbiology, School of Biological Sciences, Seoul National University, Seoul, South Korea; 3 Laboratory of Biophysics, School of Biological Sciences, Seoul National University, Seoul, South Korea; Brandeis University, UNITED STATES

## Abstract

Though nanomaterials are considered as drug carriers or imaging reagents targeting the central nervous system their cytotoxicity effect on neuronal cells has not been well studied. In this study, we treated PC12 cells, a model neuronal cell line, with a nanomaterial that is widely accepted for medical use, superparamagnetic iron oxide nanoparticles (SPIONs). Our results suggest that, after treated with SPIONs, the expression pattern of the cellular miRNAs changed widely in PC12 cells. As potential miRNA targets, NMDAR, one of the candidate mRNAs that were selected using GO and KEGG pathway enrichment, was significantly down regulated by SPIONs treatment. We further illustrated that SPIONs may induce cell death through NMDAR suppression. This study revealed a NMDAR neurotoxic effect of SPIONs and provides a reliable approach for assessing the neurocytotoxic effects of nanomaterials based on the comprehensive annotation of miRNA profiling.

## Introduction

The rapid development of nanotechnology provides a flexible platform for generating nanomaterials with specific functions for medical applications due to their unique properties. For example, nanoparticles, which usually are 10–100 nm in diameter, can easily flow through blood capillaries to reach their target sites. Nanoparticles can be technologically engineered and modified so that they carry components for medical imaging, cancer therapy or drug release [1–9]. Because of the versatile nature of the conjugate, nanomaterial has been wildly used in diagnosis and treatment. Different bulk forms can be generated by different nanotechnology procedures and may endow a given nanomaterial with new properties, in some cases involving completely unexpected physical and chemical properties [10–13]. For this reason, the de novo capabilities of nanomaterials are still under investigation. Besides the emerging applications of nanomaterials in biological systems, the cellular effects of nanomaterials are still unclear. Several studies based on the toxicity of nanomaterial in biological systems indicate the need for a new scientific discipline focused on nanotoxicity [14–18]. Due to the complexity of nanomaterials and their effects on living organisms, few studies have been able to make a robust conclusion about the cytotoxicity of certain nanomaterials. Indeed, for some nanomaterials that are suggested to have low cytotoxicity, their cellular effect and long-term safety need further inspection. Unfortunately, except for the conventional toxicity assay, there is a lack of reliable methodology for systematically assessing the overall cellular effects of specific materials.

The overwhelming majority of assays to test nanotoxicity in biological systems are performed *in vitro* with the advantages over *in vivo* studies of providing less ethical ambiguity, being easier to reproduce and carrying less expense. Conventional tests for cellular nanotoxicity include assays for cytotoxicity, genotoxicity or altered gene expression, and these assessments are carried out using standard in vitro assays, such as Northern blotting, real-time PCR, or microarray analyses [19–21]. Based on high-throughput microarray and bioinformatics analyses, gene expression profiling may provide a systematic method for examining the biocompatibility of nanomaterials. However, mRNA assays may not accurately reflect the response state of a cell due to the inescapable degradation of a portion of the mRNAs during sample preparation and the regulation of proteins through post-transcriptional mechanisms such as effects on translation.

MicroRNAs (miRNAs) are short RNA molecules working as post-transcriptional regulators by binding to complementary sequences on target mRNA transcripts. By enacting gene silencing through translational repression or target degradation, miRNAs may regulate comprehensive biological processes, including cell viability, proliferation, development and differentiation [22–26]. Methods have been developed for profiling miRNA expression, for example, the deep sequencing technique [27]. Based on the increased stability of miRNA during sample processing, miRNA expression profiling may provide a more reliable method for evaluating the biocompatibility of nanomaterial. Several studies have revealed that miRNAs is regulated in response to the cytotoxicity of nanomaterial [28–30]. Recently, miRNAs involved in the cytotoxicity of CdTe quantum dots in NIH/3T3 cells has been illustrated, demonstrating that SOLiD sequencing-based miRNA expression profiling provides a viable method for analyzing the nanomaterial cytotoxicity [31].

Engineered superparamagnetic iron oxide nanoparticles (SPIONs) can be used as advanced carriers for delivering therapeutic reagents and noninvasive magnetic resonance imaging (MRI) [32–34]. Because capable of crossing through the blood-brain barrier, SPIONs are engineered for targeted imaging and therapy for brain diseases [35, 36]. With regards to the central nervous system (CNS), the interaction between nanoparticles and neurons needs to be inspected. Several studies have investigated the effects of nanomaterials on PC12 cells, a neuroendocrine cell line with the capability of producing the neurotransmitter dopamine and activating functional metabolic pathways in response to it [37–39]; however, the comprehensive effects of nanomaterials on neurons need to be further explored. In this study, the effect of SPIONs on the PC12 cell line were systematically evaluated based on differential miRNA expression profiling analyzed with a newly modified mathematical model and web-based bioinformatics. The fact that that SPIONs may induce cell death through NMDAR suppression was also revealed for the first time by bioinformatics analysis. These results are consistent with protein electrophoresis analysis. Thus, this paper provides a systematic biological approach for evaluating nanomaterial biocompatibility with neurons.

## Methods

### 1. Preparation of nanoparticles

SPIONs were donated by Jiangsu Laboratory for Biomaterials and Devices. Transmission electron microscopy (TEM) images were taken with a Tecnai 20 microscope (Oregon, USA). The TEM samples were prepared by dropping the freshly prepared solution onto a 300-mesh carbon-coated copper grid (SPI supplies, USA). For the details of preparing SPIONs and more information of characterize of SPION, such as zeta potential and DLS data, please refer to studies reported previously[40–42].

### 2. Cell culture

Rat pheochromocytoma (PC12) cells were obtained from the cell bank of Institute of Biochemistry and Cell Biology, SIBS, CAS (Shanghai, China) andas described previously[43], were cultured in Ham’s F12K (Gibco, Canada), containing 2 mM L-glutamine (Gibco), with 15% horse serum (Gibco), 2.5% fetal bovine serum (FBS) (Gibco) and 1% penicillin/streptomycin (Gibco) at 37°C in a 5% CO_2_ incubator (Hera Cell 150, Thermo, US).

### 3. Small RNA extraction, SOLiD sequencing and Bioinformatics analysis

The cells were plated at an initial density of 1 × 10^5^ cells/ml in 35 mm culture dishes. After 24 h, they were exposed to SPIONs (214 μg/ml) for an additional 24 h. The cells were harvested with trypsin to extract the small RNAs using the mirVana miRNA isolation kit (Ambion, TX, USA) according to the manufacturer’s protocol.

The small RNAs were converted into a double-stranded cDNA library using a SOLiD Small RNA Expression Kit (Invitrogen, CA, USA), which is compatible with the Applied Biosystems SOLiD System (Life Technologies, CA, USA). The results of SOLiD sequencing were obtained in the form of nucleotide sequences and their coverage. The registered miRNAs were identified by comparing them to sequences in Genbank (www.ncbi.nlm.nih.gov/GenBank) and miRbase (http://www.mirbase.org). The dysregulated expression of miRNAs in the nanomaterial treated PC12 cells was analyzed and the comprehensive repression effect on each mRNA of the whole miRNA profile was calculated following the method reported previously [31, 44]. Finally, the significantly affected genes were assigned to either the KEGG pathways or the GO biological processes through DAVID.

### 4. Western blotting

After culturing in the indicated conditions, protein was extracted from the cells. The protein concentrations of homogenized lysates was measured by Nanodrop (Thermo, DE, USA), and 10 μg of protein extracted from cancer cells was separated on a 10% SDS-PAGE gel and transblotted onto a polyvinylidene fluoride membrane (BioRad, CA, USA). After blocking in a powdered nonfat milk solution (5% in PBS with 0.05% Tween-20 (Thermo)), the blot was incubated with polyclonal rabbit anti-rat antibodies against NMDAR2A (Abcam, Cambridge, MA), NMDAR2C (Abcam), NMDAR2D (Millipore, Billerica, MA, USA), caspase-12 (Abcam), cytochrome (Cyt)-c (Abcam), or actin (Abcam) at a 1:1000 dilution in 5% blocking solution (Abcam) overnight at 4°C. An anti-rabbit IgG antibody (Abcam) at a dilution of 1:5000 was used as a secondary antibody. Protein expression patterns were detected with an enhanced chemiluminescence kit (ECL; Amersham Bioscience/GE Healthcare, Little Chalfont, UK).

### 5. Statistical analysis

The results were presented as the mean ± standard deviation (SD). The statistical significance of the differences was tested with the Student’s t-test.In all comparisons, values of p<0.05 (*) were considered statistically significant.

## Results and Discussion

### 1. SPION treatment induces a change in the microRNA expression profile in neurons without impairing their viability

**Fig 1:**
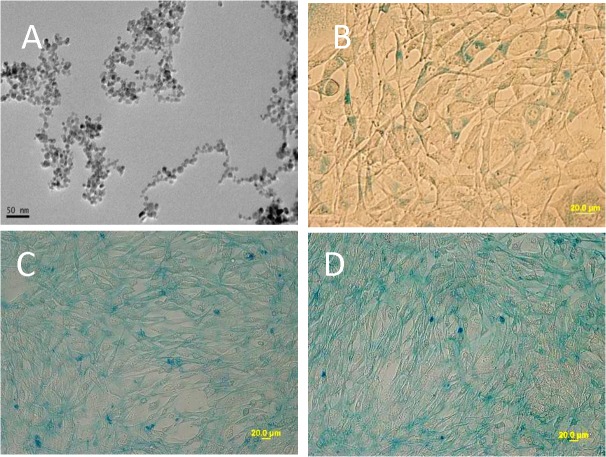
Morphology and uptake of SPIONS in PC12 cells. A confocal image of the SPIONs is shown (A). Prussian blue staining was performed to measure the uptake of SPIONs (214ug/ml) in PC12 cells at 0 (B), 12 (C) and 24 h (D). The majority of cells can incorporate SPIONs at 24 h (D).

A microscopic image of the SPIONs is shown in Fig. 1A. In order to address their cellular effect, PC12 cells were treated with 214 μg/ml of SPIONs. Prussian blue staining indicated the translocation of SPIONs into PC12 cells following 0 h (Fig. 1B), 12 h (Fig. 1C) and 24 h exposure (Fig. 1D). Results indicated that the majority of cells can incorporate SPIONs at 24 h. a significant increasing of nanoparticle uptake has not been observed after 24 h. Endocytosis have been regarded as the possible mechanism of mammalian cells for nanoparticle internalization [45]. Generally, it was thought that particles with sizes <0.5 μm is through pinocytosis, while particles with sizes >0.5μm is through phagocytosis. Since the diameter of SPIONs used in our study is about 4–7nm, it is possible that SPIONs were internalized through pinocytosis. Pinocytosis process has been further subcategorized into macropinocytosis, clathrin-dependent endocytosis, caveolin- dependent endocytosis, and clathrin- and caveolin-independent endocytosis. Intensive studies have been done for identifying the pathway of internalization of nanoparticle, however, there still no consensus about the transport mechanism due to the different cell type and different properties of nanoparticle used in the experiments [46–49]. Once nanoparticles were internalized in to the cells, biological process of the cells can be wildly changed. In this study, we concerned about the neurotoxicity effect of SPIONs [50–53].

**Fig 2:**
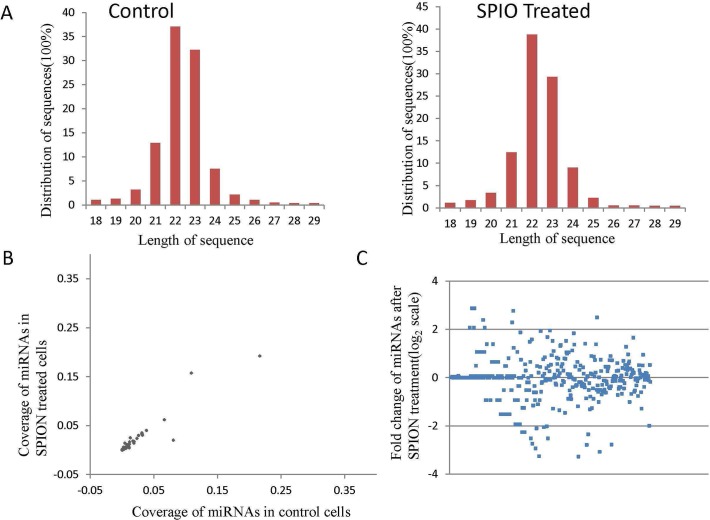
miRNA high-throughput deep sequencing for PC12 cells treated with or without SPIONs. Deep sequencing results show that the majority of the reads were 18–25nt, with the most abundant size of 22nt in both the control and SPIONs treated group (A). The abundance of many miRNAs is less than 0.1% (B). Correlation analysis between the convergence of miRNAs in SPIONs treated or non-treated cells shows that the relative amounts of some miRNAs deviated strikingly, although most miRNAs were regulated somewhat (C).

To investigate the potential neurotoxicity of SPIONs, miRNAs were extracted from PC12 cells treated with 214 μg/ml SPIONs and compared to untreated control cells. The expression patterns of miRNAs were analyzed using a high-throughput deep sequencing method. A total of tens millions SOLiD sequencing events were obtained for the miRNA samples. According to the size distribution of the sequencing reads, the majority of the miRNAs were 18–25 nt, with the most abundant size of 22 nt in both the control and SPION treated groups (Fig. 2A). After filtering the contaminated reads (those contaminated by other RNA pieces), a total of 388 miRNAs were identified with hundreds of thousands of copies in each sample. The low coverage (0.1%) of many miRNAs guaranteed the feasibility of the SOLiD sequencing data (Fig. 2B). The comparison of miRNAs in SPIONs treated and non-treated cells was analyzed. As shown, the expression of some miRNAs was obviously different in the two groups, although most miRNAs were regulated somewhat (Fig. 2C). The accuracy of SOLiD sequencing results was verified by qRT-PC, which is generally accepted as a reliable method for measuring relative expression levels of miRNAs. Correlation between the relative expression levels of miRNAs obtained with SOLiD sequencing and qRT-PC was clear and statistically consistent (data not shown).

### 2. Profiling of the comprehensive translational suppression by miRNAs after SPION treatment

**Fig 3:**
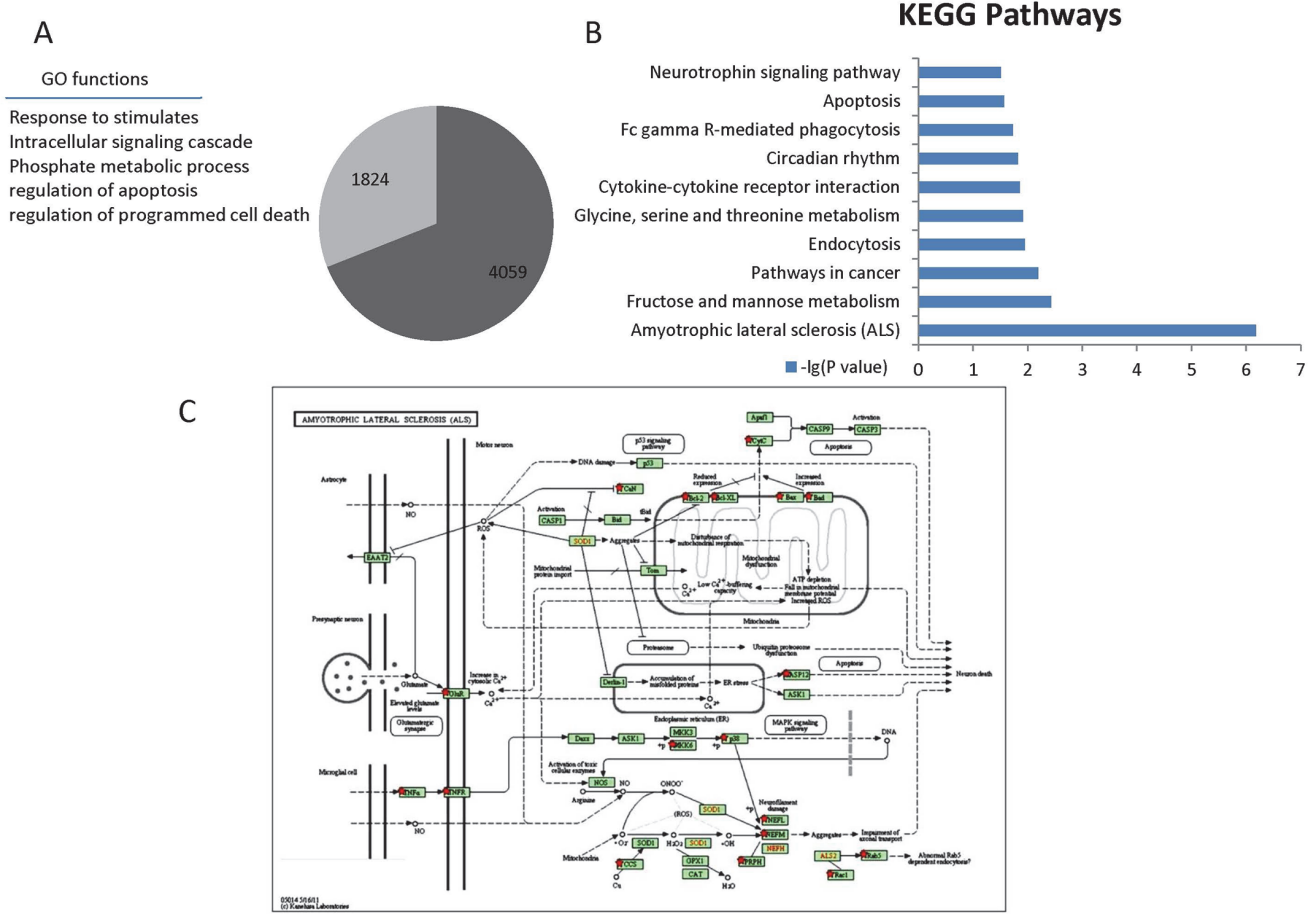
Assessment of neuronal cytotoxicity of SPIONs. Based on their miRNA target profile, 5883 mRNAs were predicted to be regulated in cells following treatment with SPIONs. Approximately 31% of the mRNA candidates were significantly regulated by nanomaterial treatment. This gene set was enriched for gene ontology (GO) functions (A). We also sorted the mRNAs into KEGG pathways through DAVID. The “Amyotrophic Lateral Sclerosis (ALS) pathway” (C) was of the most highly affected KEGG pathway with p <10^−7^(B).

With the method reported previously [44], the comprehensive suppression effects of a given miRNA profile on the translation of mRNAs can be predicted. There were 5883 mRNAs predicted to be regulated in the cells treated with SPIONs. For selecting the mRNAs that may be significantly repressed, a Z-test was performed comparing the differences between the repression of miRNAs in control cells and SPION treated cells. As a result, approximately 31% of the targeted mRNAs were predicted to be significantly regulated after nanomaterial treatment. This gene set was enriched for gene ontology (GO) functions associated with the cellular response to stimulation, stress or phosphate metabolic processes and contained multiple markers associated with cell death, apoptosis or programmed cell death (Fig. 3A).

Based on the mRNAs that were predicted to be significantly regulated by the integrated miRNA expression profile, we sought to investigate the intracellular changes in PC12 cells induced by SPION exposure. Because the KEGG pathway database records networks of molecular interactions within cells, we enriched for the mRNAs that were predicted to be significantly dysregulated by analysis of KEGG pathways through DAVID. Fig. 3B shows the KEGG pathways that were significantly affected by SPION treatment. Among these, the “Amyotrophic Lateral Sclerosis (ALS) pathway” (illustrated diagrammatically in Fig. 3C; This also can be found online: http://www.genome.jp/kegg/pathway/hsa/hsa05014.html) was predicted to be most highly affected with a *P* <10^−7^, which indicates that SPION exposure may trigger neuron degeneration pathways.

**Fig 4:**
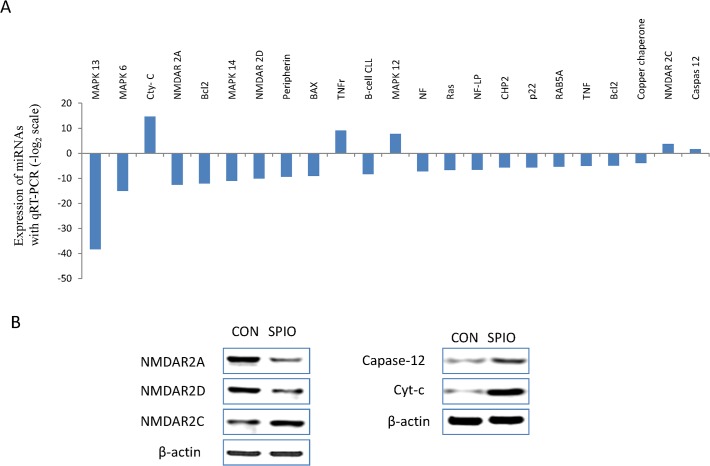
SPIONs induce neuronal death through the NMDAR pathway. A selected group of the genes predicted to be regulated by SPION treatment are shown (A). The expression of NMDAR2A and NMDAR2D were suppressed, while NMDAR2C showed a slight elevation expression pattern, which was verified by western blotting (B). The expression of apoptosis-related proteins, capase-12 and Cyt-c were also predicted to be unregulated and were verified to be increased following treatment with SPION (C). NF: Neurofilament. NF-LP: Neurofilament-light peptide.

The mRNAs within the ALS pathway are elaborated in Fig. 4A according to their calculated Z value. Within this list, p38 mitogen-activated protein family kinases were dramatically dysregulated. Mitogen-activated protein kinase 13 (MAPK13) and mitogen-activated protein kinase 14 (MAPK14) are predicted to be significantly suppressed by miRNAs compared with the control group. Given that the p38-isoform is mainly distributed in the adrenal gland and pituitary, it is not surprise that it comprises the most dramatically regulated isoform [54–56]. Interestingly, our data showed that miRNAs targeted to mitogen-activated protein kinase 12 (MAPK12), which is mainly expressed in skeleton muscle, had an increased expression pattern.

### 3. SPIONs induce neuron death through the NMDAR pathway

PC12 cells are derived from chromaffin cells of the adrenal medulla and are widely used as a model system for sympathetic ganglion-like neurons [57,58]. It has been shown that N-methyl-D-aspartate receptor (NMDAR), a glutamate receptor, is expressed on PC12 cells [59–61]. NMDAR is important for processes underlying neuronal plasticity, outgrowth and survival. The functional NMDAR receptor is assembled from an essential subunit, NMDAR subunit 1 (NMDAR1), together with one or more of a second family of subunits termed NMDAR subunits 2A-D (NMDAR2A-D) [62, 63]. As a ligand-gated ion channel, NMDAR is highly permeable to Ca^2+^. Excitatory amino acids, such as glutamate, may cause membrane depolarization and accumulation of cytosolic Ca^2+^ by enhancing cellular Ca^2+^ influx and releasing Ca^2+^ from intracellular compartments. However, several studies have illustrated that suppression of NMDAR induces the activation of a series of neurotoxic events, including cell death through the NMDAR-Caspase pathway [64–67]. Fig. 4A shows that after SPION treatment the expression of NMDAR2A and NMDAR2D was predicted to be suppressed and NMDAR2C was elevated. The apoptosis-related proteins Cyt-C and Caspase 12 are also thought to function within the NMDAR pathway of cell death (Fig. 3A). Each of these proteins was shown to follow the expected pattern of expression in response to SPION treatment (Fig. 4B). This is the first report of a role for these apoptosis-related proteins in NMDAR signaling. Taken together, these results derived from comprehensive assessment of miRNA expression profiling suggest that SPIONs may induce cell apoptosis through the NMDAR pathway in neuron cells.

## Conclusions

Although SPIONs are widely accepted as magnetic resonance imaging reagents for disease diagnosis, including brain disease, as well as drug carriers targeting the CNS, the effect of SPIONs on neuronal cell has not been well studied. To elaborate their effects on neuronal cells, the expression profiles of miRNAs in neurons were comprehensively analyzed with a method we developed. Our results suggest after PC12 was treated by SPIONs, the miRNA expression pattern was widely changed. To identify potential targets, candidate mRNAs were selected using GO and KEGG pathway enrichment. Genes related to cell death or apoptosis pathways were identified. This indicated that SPIONs may trigger neuron degeneration pathways, including the ALS pathway. Based on this comprehensive analysis, we illustrated that SPIONs may induce neuron death through the glutamate receptor, NMDAR in sympathetic ganglion-like neurons. Thus, we systematically analyzed the interaction between neurons and SPIONs, a widely used nanoparticle, by a new method based on miRNA sequencing technology and demonstrated that NDMAR may play an important role in the cell death induction by SPIONs.

## References

[1] Kircher MF, Mahmood U, King RS, Weissleder R, Josephson L (2003) A Multimodal Nanoparticle for Preoperative Magnetic Resonance Imaging and Intraoperative Optical Brain Tumor Delineation. Cancer Research 63: 8122–8125. 14678964

[2] Yang H (2010) Nanoparticle-Mediated Brain-Specific Drug Delivery, Imaging, and Diagnosis. Pharmaceutical Research 27: 1759–1771. 10.1007/s11095-010-0141-7 20593303 PMC2933363

[3] Kircher MF, de la Zerda A, Jokerst JV, Zavaleta CL, Kempen PJ, et al (2012) A brain tumor molecular imaging strategy using a new triple-modality MRI-photoacoustic-Raman nanoparticle. Nat Med 18: 829–834. 10.1038/nm.2721 22504484 PMC3422133

[4] Kingwell K (2012) Neuro-oncology: Nanoparticle imaging could guide brain tumour surgery. Nat Rev Neurol 8: 296–296. 10.1038/nrneurol.2012.84 22614851

[5] Jiang W, Xie H, Ghoorah D, Shang Y, Shi H, et al (2012) Conjugation of Functionalized SPIONs with Transferrin for Targeting and Imaging Brain Glial Tumors in Rat Model. PLoS ONE 7: e37376 10.1371/journal.pone.0037376 22615995 PMC3355132

[6] Liu Y, Fang J, Joo K-I, Wong MK, Wang P (2014) Codelivery of Chemotherapeutics via Crosslinked Multilamellar Liposomal Vesicles to Overcome Multidrug Resistance in Tumor. PLoS ONE 9: e110611 10.1371/journal.pone.0110611 25330237 PMC4201570

[7] Liu Y, Fang J, Kim Y-J, Wong MK, Wang P (2014) Codelivery of Doxorubicin and Paclitaxel by Cross-Linked Multilamellar Liposome Enables Synergistic Antitumor Activity. Molecular Pharmaceutics 11: 1651–1661. 10.1021/mp5000373 24673622 PMC4018157

[8] Schroeder BR, Ghare MI, Bhattacharya C, Paul R, Yu Z, et al (2014) The Disaccharide Moiety of Bleomycin Facilitates Uptake by Cancer Cells. Journal of the American Chemical Society 136: 13641–13656. 10.1021/ja507255g 25184545 PMC4183664

[9] Yu Z, Schmaltz RM, Bozeman TC, Paul R, Rishel MJ, et al (2013) Selective Tumor Cell Targeting by the Disaccharide Moiety of Bleomycin. Journal of the American Chemical Society 135: 2883–2886. 10.1021/ja311090e 23379863 PMC3605894

[10] Yu T, Malugin A, Ghandehari H (2011) Impact of Silica Nanoparticle Design on Cellular Toxicity and Hemolytic Activity. ACS Nano 5: 5717–5728. 10.1021/nn2013904 21630682 PMC3238493

[11] Soonhyang P, Hicham C, Jody W, Jay LN (2011) Antimicrobial activity and cellular toxicity of nanoparticle–polymyxin B conjugates. Nanotechnology 22: 185101 10.1088/0957-4484/22/18/185101 21415471

[12] Khamwannah J, Zhang Y, Young Noh S, Kim H, Frandsen C, et al (2012) Enhancement of dye sensitized solar cell efficiency by composite TiO2 nanoparticle/8 nm TiO2 nanotube paper-like photoelectrode. Nano Energy 1: 411–417. 10.1016/j.bios.2012.05.003 22651966

[13] Zhu K, Guo C, Lai H, Yang W, Wang C (2012) Novel hyperbranched polyamidoamine nanoparticle based gene delivery: Transfection, cytotoxicity and in vitro evaluation. International Journal of Pharmaceutics 423: 378–383. 10.1016/j.ijpharm.2011.12.030 22209826

[14] Chou LYT, Chan WCW (2012) Nanotoxicology: No signs of illness. Nat Nano 7: 416–417. 10.1038/nnano.2012.110 22760019

[15] Tavares A, Louro H, Antunes S, Quarre S, Simar S, et al (2014) Genotoxicity evaluation of nanosized titanium dioxide, synthetic amorphous silica and multi-walled carbon nanotubes in human lymphocytes. Toxicol In Vitro 28: 60–69. 10.1016/j.tiv.2013.06.009 23811260

[16] Manke A, Luanpitpong S, Dong C, Wang L, He X, et al (2014) Effect of Fiber Length on Carbon Nanotube-Induced Fibrogenesis. International Journal of Molecular Sciences 15: 7444–7461. 10.3390/ijms15057444 24786100 PMC4057682

[17] Liu Y, Rohrs J, Wang P (2014) Development and challenges of nanovectors in gene therapy. Nano LIFE 4.

[18] Siegrist K, Reynolds S, Kashon M, Lowry D, Dong C, et al (2014) Genotoxicity of multi-walled carbon nanotubes at occupationally relevant doses. Particle and Fibre Toxicology 11: 6 10.1186/1743-8977-11-6 24479647 PMC3923549

[19] Patil G, Khan MI, Patel DK, Sultana S, Prasad R, et al (2012) Evaluation of cytotoxic, oxidative stress, proinflammatory and genotoxic responses of micro- and nano-particles of dolomite on human lung epithelial cells A549. Environmental Toxicology and Pharmacology 34: 436–445. 10.1016/j.etap.2012.05.014 22785077

[20] Park MVDZ, Verharen HW, Zwart E, Hernandez LG, van Benthem J, et al (2011) Genotoxicity evaluation of amorphous silica nanoparticles of different sizes using the micronucleus and the plasmid lacZ gene mutation assay. Nanotoxicology 5: 168–181. 10.3109/17435390.2010.506016 20735203

[21] Fu J, Rong G, Deng Y (2012) Mammalian Cell Cytotoxicity and Genotoxicity of Metallic Nanoparticles. Advanced Science Letters 5: 294–298.

[22] Sayed D, Abdellatif M (2011) MicroRNAs in Development and Disease. Physiological Reviews 91: 827–887. 10.1152/physrev.00006.2010 21742789

[23] Perruisseau-Carrier C, Jurga M, Forraz N, McGuckin C (2011) miRNAs Stem Cell Reprogramming for Neuronal Induction and Differentiation. Molecular Neurobiology 43: 215–227. 10.1007/s12035-011-8179-z 21541853

[24] Ruan J, Lou S, Dai Q, Mao D, Ji J, et al. (9000) Tumor suppressor miR-181c attenuates proliferation, invasion, and self-renewal abilities in glioblastoma. NeuroReport Publish Ahead of Print: 10.1097/WNR.0000000000000302. 10.1097/WNR.0000000000000302 25494473

[25] Izarra A, Moscoso I, Cañón S, Carreiro C, Fondevila D, et al. (2014) miRNA-1 and miRNA-133a are involved in early commitment of pluripotent stem cells and demonstrate antagonistic roles in the regulation of cardiac differentiation. Journal of Tissue Engineering and Regenerative Medicine: n/a-n/a. 10.1002/term.1977 25492026

[26] Sun K-T, Chen MYC, Tu M-G, Wang IK, Chang S-S, et al. MicroRNA-20a regulates autophagy related protein-ATG16L1 in hypoxia-induced osteoclast differentiation. Bone. 10.1016/j.bone.2014.11.026 25485521

[27] Creighton CJ, Reid JG, Gunaratne PH (2009) Expression profiling of microRNAs by deep sequencing. Briefings in Bioinformatics 10: 490–497. 10.1093/bib/bbp019 19332473 PMC2733187

[28] Mahmood M, Li Z, Casciano D, Khodakovskaya MV, Chen T, et al (2011) Nanostructural materials increase mineralization in bone cells and affect gene expression through miRNA regulation. Journal of Cellular and Molecular Medicine 15: 2297–2306. 10.1111/j.1582-4934.2010.01234.x 21143388 PMC3822941

[29] Suh WH, Suslick KS, Stucky GD, Suh Y-H (2009) Nanotechnology, nanotoxicology, and neuroscience. Progress in Neurobiology 87: 133–170. 10.1016/j.pneurobio.2008.09.009 18926873 PMC2728462

[30] Mellado-Gil J, Rosa TC, Demirci C, Gonzalez-Pertusa JA, Velazquez-Garcia S, et al (2011) Disruption of Hepatocyte Growth Factor/c-Met Signaling Enhances Pancreatic beta-Cell Death and Accelerates the Onset of Diabetes. Diabetes 60: 525–536. 10.2337/db09-1305 20980460 PMC3028352

[31] Li S, Wang H, Qi Y, Tu J, Bai Y, et al (2011) Assessment of nanomaterial cytotoxicity with SOLiD sequencing-based microRNA expression profiling. Biomaterials 32: 9021–9030. 10.1016/j.biomaterials.2011.08.033 21889204

[32] Patel D, Kell A, Simard B, Deng J, Xiang B, et al (2010) Cu2+-labeled, SPION loaded porous silica nanoparticles for cell labeling and multifunctional imaging probes. Biomaterials 31: 2866–2873. 10.1016/j.biomaterials.2009.12.025 20053440

[33] Mi Kyung Y, Jinho P, Yong Yeon J, Woo Kyung M, Sangyong J (2010) Integrin-targeting thermally cross-linked superparamagnetic iron oxide nanoparticles for combined cancer imaging and drug delivery. Nanotechnology 21: 415102 10.1088/0957-4484/21/41/415102 20852354

[34] Veiseh O, Gunn JW, Zhang M (2010) Design and fabrication of magnetic nanoparticles for targeted drug delivery and imaging. Advanced Drug Delivery Reviews 62: 284–304. 10.1016/j.addr.2009.11.002 19909778 PMC2827645

[35] Bigini P, Diana V, Barbera S, Fumagalli E, Micotti E, et al (2012) Longitudinal Tracking of Human Fetal Cells Labeled with Super Paramagnetic Iron Oxide Nanoparticles in the Brain of Mice with Motor Neuron Disease. PLoS ONE 7: e32326 10.1371/journal.pone.0032326 22384217 PMC3288077

[36] Carp SA, Kim YR, Dai G, Boas DA, Franceschini MA. Validation of Optical Measurements of Cerebral Blood Flow and Volume with SPION and ASL fMRI. OSA Technical Digest (CD); 2008. Optical Society of America. pp. BMD2.

[37] Rivet CJ, Yuan Y, Borca-Tasciuc D-A, Gilbert RJ (2011) Altering Iron Oxide Nanoparticle Surface Properties Induce Cortical Neuron Cytotoxicity. Chemical Research in Toxicology 25: 153–161. 10.1021/tx200369s 22111864 PMC4183191

[38] Hu Y-L, Gao J-Q (2010) Potential neurotoxicity of nanoparticles. International Journal of Pharmaceutics 394: 115–121. 10.1016/j.ijpharm.2010.04.026 20433914

[39] Pisanic Ii TR, Blackwell JD, Shubayev VI, Fiñones RR, Jin S (2007) Nanotoxicity of iron oxide nanoparticle internalization in growing neurons. Biomaterials 28: 2572–2581. 17320946 10.1016/j.biomaterials.2007.01.043

[40] He S, Feng Y, Gu N, Zhang Y, Lin X (2011) The effect of γ-Fe2O3 nanoparticles on Escherichia coli genome. Environmental Pollution 159: 3468–3473. 10.1016/j.envpol.2011.08.024 21917366

[41] Sun J, Liu X, Huang J, Song L, Chen Z, et al (2014) Magnetic assembly-mediated enhancement of differentiation of mouse bone marrow cells cultured on magnetic colloidal assemblies. Sci Rep 4. 10.1038/srep05125 PMC4038806 24874764

[42] Zhang S, Chen X, Gu C, Zhang Y, Xu J, et al (2009) The Effect of Iron Oxide Magnetic Nanoparticles on Smooth Muscle Cells. Nanoscale Research Letters 4: 70–77.

[43] Wang G, Qi C, Fan G-H, Zhou H-Y, Chen S-D PACAP protects neuronal differentiated PC12 cells against the neurotoxicity induced by a mitochondrial complex I inhibitor, rotenone. FEBS Letters 579: 4005–4011. 16004991 10.1016/j.febslet.2005.06.013

[44] Sun B, Yang F, Hu F-H, Huang N-P, Xiao Z-D (2013) Comprehensive annotation of microRNA expression profiles. BMC Genetics 14: 120 10.1186/1471-2156-14-120 24359251 PMC3878191

[45] Gu J, Xu H, Han Y, Dai W, Hao W, et al (2011) The internalization pathway, metabolic fate and biological effect of superparamagnetic iron oxide nanoparticles in the macrophage-like RAW264.7 cell. Science China Life Sciences 54: 793–805. 10.1007/s11427-011-4215-5 21922429

[46] Raynal I, Prigent P, Peyramaure S, Najid A, Rebuzzi C, et al (2004) Macrophage Endocytosis of Superparamagnetic Iron Oxide Nanoparticles: Mechanisms and Comparison of Ferumoxides and Ferumoxtran-10. Investigative Radiology 39: 56–63. 14701989 10.1097/01.rli.0000101027.57021.28

[47] Cai H, Yao P (2014) Gold nanoparticles with different amino acid surfaces: Serum albumin adsorption, intracellular uptake and cytotoxicity. Colloids and Surfaces B: Biointerfaces 123: 900–906. 10.1016/j.colsurfb.2014.10.042 25466455

[48] Ferrari R, Lupi M, Colombo C, Morbidelli M, D’Incalci M, et al (2014) Investigation of size, surface charge, PEGylation degree and concentration on the cellular uptake of polymer nanoparticles. Colloids and Surfaces B: Biointerfaces 123: 639–647. 10.1016/j.colsurfb.2014.10.003 25456985

[49] Bannunah AM, Vllasaliu D, Lord J, Stolnik S (2014) Mechanisms of Nanoparticle Internalization and Transport Across an Intestinal Epithelial Cell Model: Effect of Size and Surface Charge. Molecular Pharmaceutics 11: 4363–4373. 10.1021/mp500439c 25327847

[50] Borisova T, Nazarova A, Dekaliuk M, Krisanova N, Pozdnyakova N, et al. Neuromodulatory properties of fluorescent carbon dots: Effect on exocytotic release, uptake and ambient level of glutamate and GABA in brain nerve terminals. The International Journal of Biochemistry & Cell Biology. 10.1016/j.biocel.2014.11.016 25486182

[51] Gomes T, Chora S, Pereira CG, Cardoso C, Bebianno MJ (2014) Proteomic response of mussels Mytilus galloprovincialis exposed to CuO NPs and Cu2+: An exploratory biomarker discovery. Aquatic Toxicology 155: 327–336. 10.1016/j.aquatox.2014.07.015 25089921

[52] Fang Q, Shi X, Zhang L, Wang Q, Wang X, et al (2015) Effect of titanium dioxide nanoparticles on the bioavailability, metabolism, and toxicity of pentachlorophenol in zebrafish larvae. Journal of Hazardous Materials 283: 897–904. 10.1016/j.jhazmat.2014.10.039 25464334

[53] Huang Y, Lü X, Qu Y, Yang Y, Wu S (2015) MicroRNA sequencing and molecular mechanisms analysis of the effects of gold nanoparticles on human dermal fibroblasts. Biomaterials 37: 13–24. 10.1016/j.biomaterials.2014.10.042 25453934

[54] Roberson MS, Zhang T, Li HL, Mulvaney JM (1999) Activation of the p38 Mitogen-Activated Protein Kinase Pathway by Gonadotropin-Releasing Hormone. Endocrinology 140: 1310–1318. 10067858 10.1210/endo.140.3.6579

[55] Hu MC-T, Wang Y-p, Mikhail A, Qiu WR, Tan T-H (1999) Murine p38-δ Mitogen-activated Protein Kinase, a Developmentally Regulated Protein Kinase That Is Activated by Stress and Proinflammatory Cytokines. Journal of Biological Chemistry 274: 7095–7102. 10066767 10.1074/jbc.274.11.7095

[56] Efimova T (2010) p38δ mitogen-activated protein kinase regulates skin homeostasis and tumorigenesis. Cell Cycle 9: 498–505. 20090411 10.4161/cc.9.3.10541

[57] Grau C, Greene L (2012) Use of PC12 Cells and Rat Superior Cervical Ganglion Sympathetic Neurons as Models for Neuroprotective Assays Relevant to Parkinson’s Disease In: Skaper SD, editor. Neurotrophic Factors: Humana Press pp. 201–211. 10.1007/978-1-61779-536-7_18 PMC3678375 22367813

[58] Johnson EM, Deckwerth TL, Deshmukh M (1996) Neuronal Death in Developmental Models: Possible Implications in Neuropathology. Brain Pathology 6: 397–409. 8944313 10.1111/j.1750-3639.1996.tb00872.x

[59] Du J, Chang J, Guo S, Zhang Q, Wang Z (2009) ApoE 4 reduces the expression of Aβ degrading enzyme IDE by activating the NMDA receptor in hippocampal neurons. Neuroscience Letters 464: 140–145. 10.1016/j.neulet.2009.07.032 19616072

[60] Ishima T, Hashimoto K (2012) Potentiation of Nerve Growth Factor-Induced Neurite Outgrowth in PC12 Cells by Ifenprodil: The Role of Sigma-1 and IP_3_ Receptors. PLoS ONE 7: e37989 10.1371/journal.pone.0037989 22655093 PMC3360021

[61] Kawakami Z, Kanno H, Ikarashi Y, Kase Y (2011) Yokukansan, a kampo medicine, protects against glutamate cytotoxicity due to oxidative stress in PC12 cells. Journal of Ethnopharmacology 134: 74–81. 10.1016/j.jep.2010.11.063 21130853

[62] Saito Y, Tsuzuki K, Yamada N, Okado H, Miwa A, et al (2003) Transfer of NMDAR2 cDNAs increases endogenous NMDAR1 protein and induces expression of functional NMDA receptors in PC12 cells. Molecular Brain Research 110: 159–168. 12591153 10.1016/s0169-328x(02)00548-x

[63] Michaelis EK (1998) Molecular biology of glutamate receptors in the central nervous system and their role in excitotoxicity, oxidative stress and aging. Progress in Neurobiology 54: 369–415. 9522394 10.1016/s0301-0082(97)00055-5

[64] Takadera T, Matsuda I, Ohyashiki T (1999) Apoptotic Cell Death and Caspase-3 Activation Induced by N-Methyl-d-Aspartate Receptor Antagonists and Their Prevention by Insulin-Like Growth Factor I. Journal of Neurochemistry 73: 548–556. 10428050 10.1046/j.1471-4159.1999.0730548.x

[65] Takadera T, Fujibayashi M, Kaniyu H, Sakota N, Ohyashiki T (2007) Caspase-dependent Apoptosis Induced by Thapsigargin was Prevented by Glycogen Synthase Kinase-3 Inhibitors in Cultured Rat Cortical Neurons. Neurochemical Research 32: 1336–1342. 17401651 10.1007/s11064-007-9310-4

[66] Adams SM, de Rivero Vaccari JC, Corriveau RA (2004) Pronounced Cell Death in the Absence of NMDA Receptors in the Developing Somatosensory Thalamus. The Journal of Neuroscience 24: 9441–9450. 15496680 10.1523/JNEUROSCI.3290-04.2004 PMC6730102

[67] Drechsel D, Estévez A, Barbeito L, Beckman J (2012) Nitric Oxide-Mediated Oxidative Damage and the Progressive Demise of Motor Neurons in ALS. Neurotoxicity Research 22: 251–264. 22488161 10.1007/s12640-012-9322-y PMC4145402

